# Numerical simulation and parameter optimization of earth auger in hilly area using EDEM software

**DOI:** 10.1038/s41598-022-23833-2

**Published:** 2022-11-14

**Authors:** Guofu Wang, Wei Zhang, Min Ji, Hu Miao, Zheng Jin

**Affiliations:** Research Institute of Wood Industry, Chinese Academy of Forestry, Beijing, 100091 China

**Keywords:** Mechanical engineering, Environmental sciences, Forestry

## Abstract

Digging in hilly regions is an important measure to promote afforestation on difficult sites. In view of the working conditions to build fish-scale pit on slope, the auger mechanism of soil lifting and throwing was investigated in this study. This study utilized EDEM software to establish the operation model of the earth auger and conduct DEM (Discrete Element Method) virtual simulation experiments. A quadratic rotating orthogonal center combination test was implemented by setting the efficiency of conveying-soil (*Y*_*1*_) and the distance of throwing-soil (*Y*_*2*_) as the evaluation indices. Variance analysis and response surface optimization were performed on the virtual experimental data. The results indicated that the weight of the factors affecting the *Y*_*1*_ and *Y*_*2*_, were feeding speed > helix angle > rotating speed > slope angle, and slope auger > rotating speed > feeding speed > helix angle. The optimal parameter combination of each influencing factor was obtained. Among them, when the slope preparation was required, the optimal operating parameter combination of the auger was: Slope of 26.467°, Helix angle of 21.567°, Feeding speed of 0.1 m/s, Rotating speed of 67.408 r/min. This research provides theoretical references for the design optimization of the earth auger in hilly regions.

## Introduction

In the process of vigorously promoting large-scale land greening in the whole society, the main problem is that, at this stage, the terrain of forestry areas to be developed is complex, the slope changes are diverse, and the afforestation conditions are difficult. Afforestation mechanization level is very low, which limited the afforestation scale expansion speed.

Cavernous soil preparation, also known as pit digging, is one of the essential links in the process of afforestation. It is widely used in the forestry production and operation processes such as tree planting, soil loosing, and deep fertilization^1^. At this stage, the developed earth auger has good adaptability in plain areas and has been widely popularized^2,3^. For hilly and mountainous areas with complex terrain, the existing augers have problems of low efficiency and low safety factor in the application process^4^.

**Figure 1:**
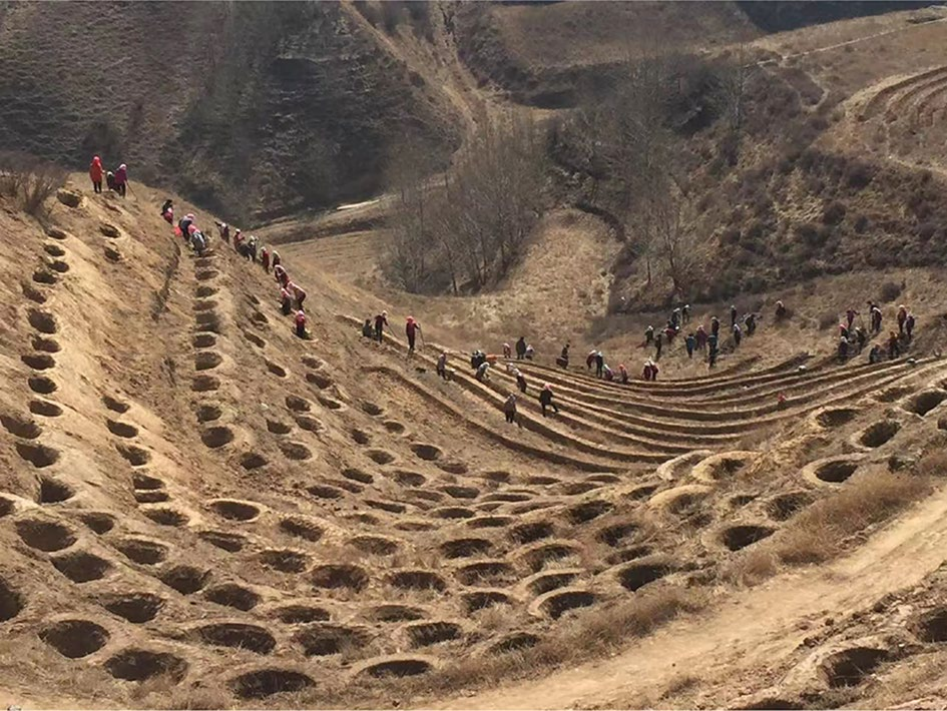
Fish-scale pit type planting woodland site.

In the afforestation operation regulations, in order to overcome the unadapt ability of earth auger and other machines and tools to hilly regions, it would be solved by carrying out level bench land preparation on the slope in advance^5^. However, the land preparation work is heavy and the original landform is seriously damaged. On the other hand, due to the narrow regional space and complex terrain, large machines cannot carry out land preparation. Horizontal land preparation is obviously not the most efficient way for tree planting^6^. When planting trees are on the slope, the shaping of fish-scale pits is one of the effective ways to conserve water and soil. The fan-shaped soil collection peak after digging on the slope has the same shape as the fish-scale pit, as shown in Fig. 1. After shaping the soil shape, it only needs the manual reinforcement^7,8^. By investigating the technology of artificial shaping fish-scale pit, this study explores the mechanized digging operation on the slope to provide helps for shaping fish scale pits.

In the 1870s, the research on the mechanism of earth auger has begun. Lian et al. conducted early research and summary on the design theory of auger. So far, many empirical formulas have been used as a reference for researchers^9,10^. Scholars, Macphersonet et al., respectively investigated the operation power consumption and bending-torsional vibration of drill bits, which contributed to the design and innovation of excavators^11,12^. In recent years, in order to solve the problems of blockage and excessive backfill rate in the process of soil transportation, many experts have used MATLAB, ADAMS, ANSYS and other simulation analysis software to analyze the statics and dynamics of the auger^13–15^.

The spatial displacement and fluctuation of soil and the interaction mechanism between soil-soil and soil-tool are the key factors affecting the energy consumption and operation effect of earth auger during the process of cutting and transporting soil. Although these studies are important for auger design and parameter optimization, they are rarely documented and published. Therefore, it is particularly important to investigate the continuous process mechanism of cutting-transportation and the dynamic response of soil.

Mustafa, Kojo, Wang and other experts applied the discrete element method to simulate the interaction between tillage components and soil, such as scarifier, rotary cultivator, plow, etc. The distribution of stress and strain in soil, dynamic soil response (such as soil displacement) and physical parameters in soil machine interface are obtained (in particular, draught, and vertical forces, energy consumption, etc.)^16–18^. DEM is one of the commonly used numerical methods in the modeling and full simulation of farming process (such as pit excavation)^19^. Jin et al., using EDEM, investigated the spiral soil-fertilizer mixing equipment, analyzed the uniformity of soil-fertilizer mixing, and obtained the best mixing operation parameters^20^. Therefore, in this study, the soil and slope modeling are developed using EDEM, and the process of auger cutting and transporting soil on slope is simulated. Through the simulation results, the dynamic characteristics of soil are analyzed, and the structural parameters and operation parameters of auger are optimized.

## Material and methods

### Working principle

**Figure 2:**
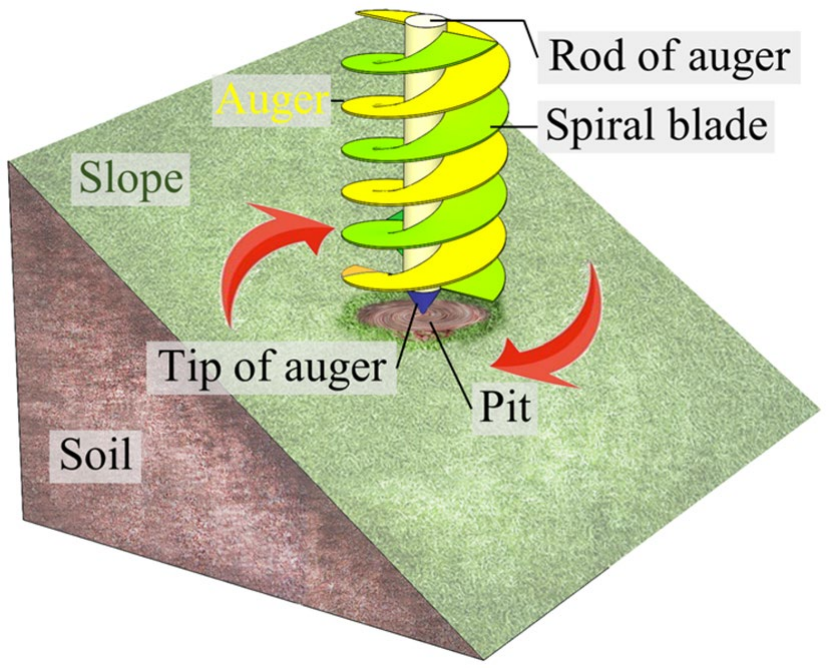
Model of auger working on slope.

Figure 2 illustrates the model of auger working on slope. The earth auger consists of spiral blades, rot and tip of auger, along with other key components. The soil is cut by spiral blades and carried out by the pit to form a cylindrical pit body^20^.

The process of soil movement on the surface of spiral blades can be obtained by observing the phenomena of field pre-experiment and virtual simulation. Soil drilling can be divided into three working processes according to the depth of the auger feeding.

The first process is the cutting slope. The two spiral blades alternately cut the lifted soil. The first possibility is that the soil leaves the spiral blades directly by the centrifugal force, completing the projectile motion and reaching the ground surface. When the soil cutting end of the auger leaves the high-altitude side of the slope and enters the air, the soil slides down to the ground surface along the blades surface by its gravity.

The second process is the deeper digging process. The cutting side end of the spiral blade is completely immersed in the soil and continuously cutting the soil. When the soil reaches the surface, most of it drains out of the pit on the high-elevation side due to different pit wall heights at the pit mouth. A preliminary fan-shaped soil collection peak is generated.

The third process is the dig of a pit similar to in the plain regions. When the height of the soil collection peak on the low altitude side is accumulated to be flush with that on the high-altitude side, the soil would be evenly sprinkled after reaching the pit mouth to form a horizontal circular soil collection peak pit mouth.

**Figure 3:**
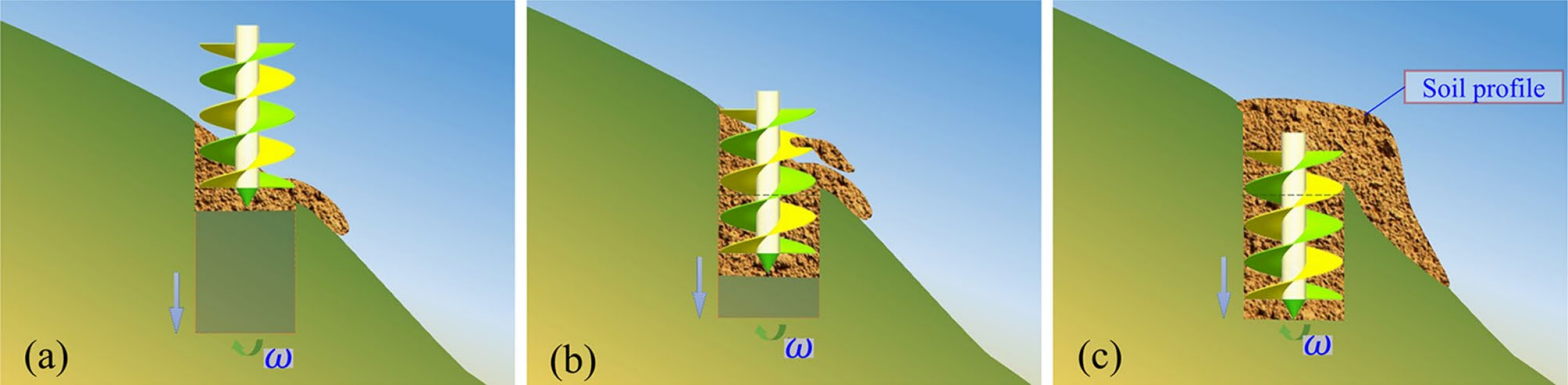
Diagram of dig process. (**a**) The first process. (**b**) The second process. (**c**) The third process.

It can be concluded that in terms of soil movement and distribution, auger operations in hilly areas are different from those in plain areas, as shown in Fig. 3. Due to the existence of slope, there are following differences in the digging process. The cutting ends of the two spiral blades break the soil alternately during the slope cutting process. The shape of the pit below the ground is an irregular cylinder, and the movement of soil is not uniform affected by the pit wall. At a certain instant, the soil on the auger is unevenly distributed, with more distribution on the higher elevation side. After the soil reaches the hill surface, it would move along the surface to the low altitude, forming a fan-shaped soil collection peak.

According to the pre-experiment, the bottom area and height parameters of fan-shaped soil collection peak are very important to the construction of fish-scale pit. If the bottom area of the soil collection peak is too large, the surface soil layer would be too thin, and it will be difficult to collect the soil. Poor discharge performance (too much soil in the pit) results in too little surface soil volume.

**Figure 4:**
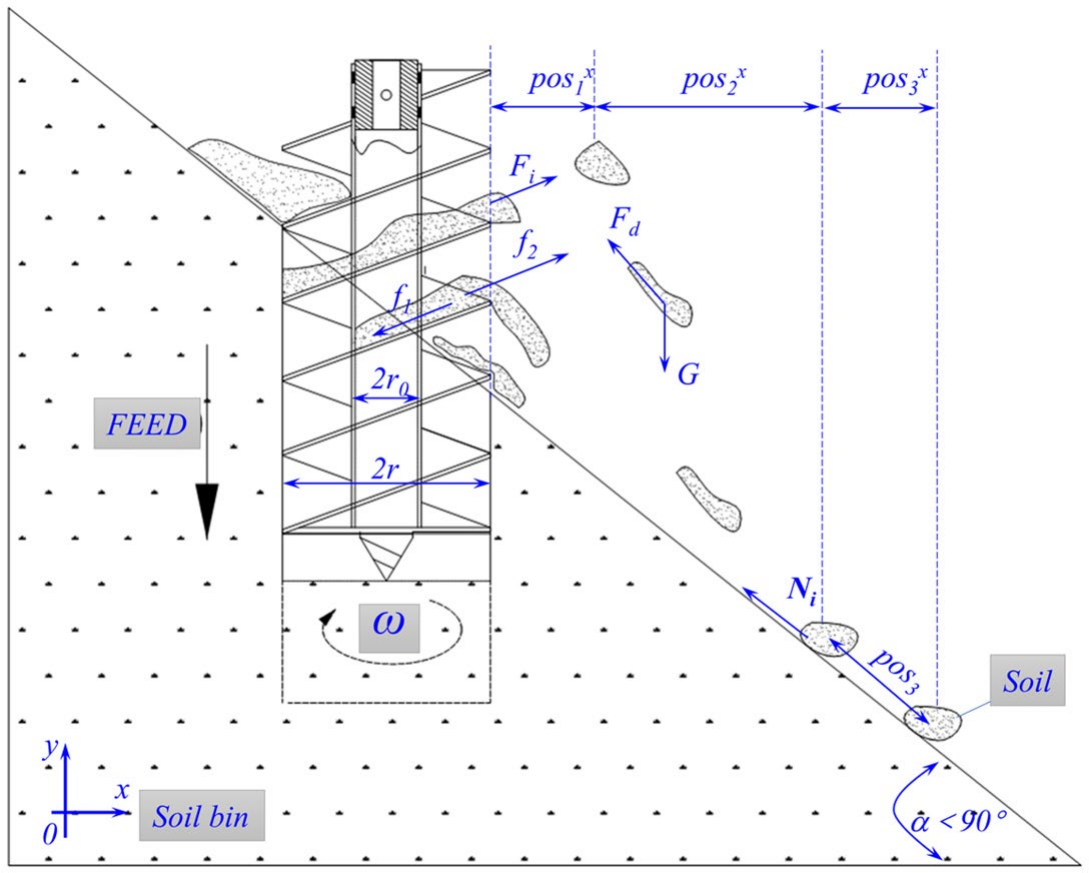
Schematic diagram of soil throwing process.

The maximum distance of throwing-soil depends mainly on the projectile motion. The soil slides down a certain distance, and then stops moving under the action of friction, as shown in Fig. 4. According to this movement process, the distance throwing-soil can be deduced, as shown in Eqs. (1)–(12).

According to the momentum theorem, it can be deduced that the absolute velocity^21^ of the soil when leaving the spiral blades as follow. The Explanation of the symbols as show in “Table of Appendix”.

$${\nu}_{0}=\frac{{\int }_{t}^{{t}_{2}}\left[mr{\left(\omega -\frac{{\nu}_{r}\mathrm{cos}\beta }{r}\right)}^{2}-\sum_{j=1}^{4}{\int }_{{\varphi }_{1}}^{{\varphi }_{2}}2{N}_{\dot{j}}{\mu }_{j}(r-{r}_{0})d\varphi +{f}_{1}+{f}_{2}\right]{d}_{t}}{m}+{\nu}_{a}$$

With

1 $${\nu}_{a}=\frac{(r-{r}_{0})\omega b}{2PC\mathrm{sin}\beta }\left[AB-\sqrt{(A{B)}^{2}-4C\left({A}^{2}\varphi -\frac{E}{{N}_{j}}\right)}\right]$$

After the soil leaves the spiral blades, it is mainly affected by gravity *G* = *mg* and air resistance ${F}_{d}=km \nu$ influence. According to the differential equation of motion, the formula is obtained as follows. The Explanation of the symbols as show in “Table of Appendix”.

Up projectile motion of soil:

2 $${\nu}_{1}^{x}={\nu}_{0}{e}^{-kt}$$

3 $${\nu}_{1}^{y}={\mathrm{e}}^{kt}{\nu}_{0}^{y}+\frac{g}{k}\left({\mathrm{e}}^{kt}-1\right)$$

4 $${pos}_{1}^{x}={\nu}_{0}^{x}\left(1-{e}^{-kt}\right)/\mu $$

5 $${pos}_{1}^{y}=\frac{\left({\nu}_{0}^{y}+g\right)\left({\mathrm{e}}^{kt}-1\right)-gt}{k}$$

Downward projectile motion of soil:

6 $${\nu}_{2}^{x}={\nu}_{1}^{x}{e}^{-kt}$$

7 $${\nu}_{2}^{y}=g\left({e}^{-kt}-1\right)/k$$

8 $${pos}_{2}^{x}={\nu }_{0}\left(1-{e}^{-kt}\right)/k$$

9 $${pos}_{2}^{y}=\frac{gt}{k}-\frac{g}{{k}^{2}}\left(1-{e}^{-kt}\right)$$

The soil slides down on the slope:

10 $${\nu}_{t}=\frac{{\nu}_{2}e{Y}_{2}}{\sqrt{{X}_{2}^{2}+{Y}_{2}^{2}}}$$

11 $${pos}_{3}=\frac{{\nu}_{t}^{2}m\mathrm{sin}\alpha }{2\left(mg-{\mu }_{1}{F}_{N}\mathrm{sin}\alpha \right)}$$

The distance of throwing-soil:

12 $$S=\frac{{pos}_{1}+{pos}_{2}}{cos\alpha }+{pos}_{3}$$

The analysis was performed without considering the material of the auger and the size of the tip, rod of the auger. According to the above formula, the distance of throwing-soil is mainly related to the surface slope, the helix angle of auger, the rotational speed of auger, and the air resistance. Under the same conditions, the greater the surface slope, the longer the time of soil throwing movement stage. The rotational speed and helix angle of auger are mainly related to the centrifugal force, which determines the initial velocity (kinetic energy) of the projectile motion.

The process of soil movement between the spiral blades was temporarily ignored. Focus on the process of dig the soil in the pit and the process discharge the soil outside of the pit. The important condition to prevent the soil from clogged in the space composed of spiral blades is that the process of digging and discharging soil is continuous. When the digging depth reaches *H*_1_, after the auger has rotated through the angle $\varphi$, the amount of soil at each position should meet the following conditions, expressed in Eq. (13).

13 $${K}_{1}{Q}_{0}+{k}_{2}{Q}_{1}-{K}_{3}{Q}_{2}\le 0.5{Q}_{3}$$

In Eq. (13), the following relations are also included as shown in Eqs. (14)–(17):

14 $${Q}_{0}=2r\left(\pi {H}_{1}-r\mathrm{tan}\beta \right)$$

$${Q}_{1}=\frac{\left(r-{r}_{0}\right)\left(\sqrt{{\left(\pi r\right)}^{2}+{{H}_{1}}^{2}}+\sqrt{{\left(\pi {r}_{0}\right)}^{2}+{{H}_{1}}^{2}}\right)h}{2}$$

with

15 $$h=\frac{2\pi {V}_{r}\varphi \mathrm{sin}\beta -S\varphi \omega }{2\pi \omega }$$

16 $${Q}_{2}={\delta }^{2}\left(r-{r}_{0}\right)\left(\frac{\omega {V}_{0}^{2}\mathrm{sin}\beta -g\varphi }{{\omega }^{3}}\right)$$

17 $${Q}_{3}=\left(r-{r}_{0}\right)\left\{H\pi \left(r+{r}_{0}\right)-\frac{1}{z}d\left(\sqrt{{\left(\pi r\right)}^{2}+{H}^{2}}+\sqrt{{\left(\pi {r}_{0}\right)}^{2}+{H}^{2}}\right)\right\}$$

According to references and Eqs. (13)–(17), the thickness of the soil *h* would affect the interaction of the drilling forces and the size of the soil movement space^22^. For *Q*_1_, the value is mainly related to the thickness h of the soil dug per unit time. The value should be increased as much as possible to improve the efficiency of digging. However, if h is too large, the congestion would occur due to space limitation of two spiral blades *Q*_3_.

As for *Q*_2_, the smoothness of the discharge-soil determines the steady supply of the subsequent soil force and the size of space of auger. To avoid clogging, *Q*_2_ should be increased as much as possible. For *Q*_2_, the value is mainly related to the velocity *v*_0_, when the soil reaches the upper edge of the pit opening and leaves the spiral blade.

For *Q*_3_, the auger in the deep digging process generally uses a double-headed spiral blade with better stability. The space of soil movement on the double blade is half of the single. Therefore, if the soil blocks are too thick, the upper surface of the soil is likely to touch the lower surface of the spiral blade, which is not conducive to soil improvement.

In conclusion, the performance of auger working on the slope can be evaluated by monitoring the efficiency of conveying-soil and the distance of throwing-soil.

### Establishment of EDEM simulation model

#### DEM parameters and virtual soil bin

**Figure 5:**
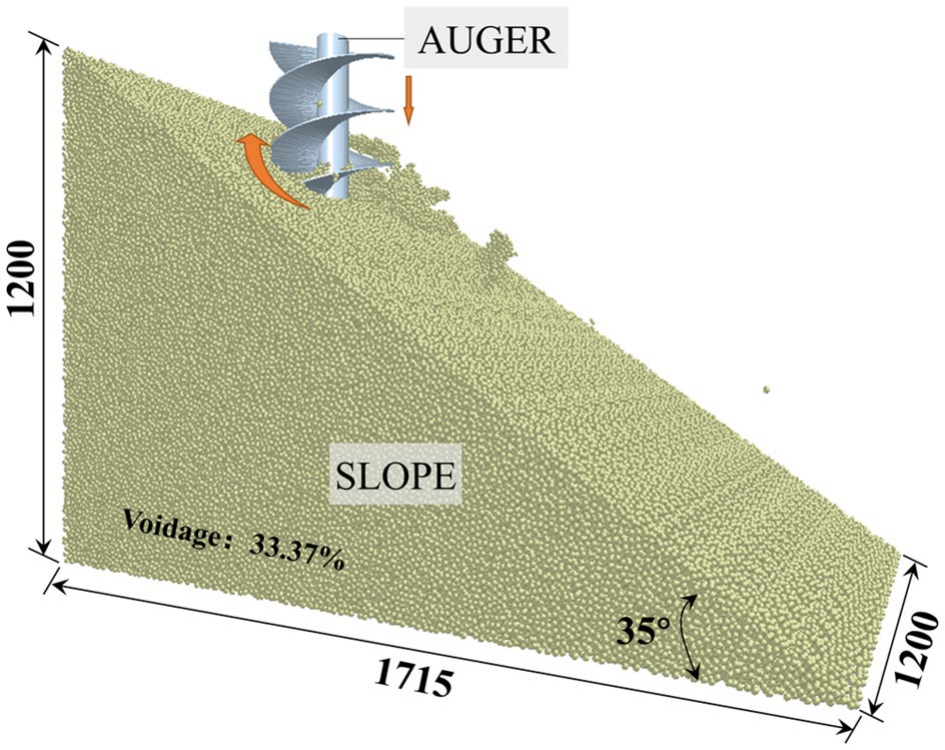
Description of virtual soil bin (Take 35° for example. All dimensions are in mm).

The effect of auger geometric features and operating parameters on the performance was evaluated by simulating the operation of the auger in a virtual soil bin using DEM, as shown in Fig. 5. The virtual soil bin was filled with spherical particles of nominal radius 7 mm. Input parameters used to describe the DEM particles and tool material properties are presented in Table 1^17,23^.

**Table 1:** Material properties of soil and tool.

Parameter	Soil	Tool
Particle diameter (mm)	7	–
Contact radius (mm)	8.5	–
Particle density (kg/m^3^)	1350	7860
Shear modulus (Pa)	1 × 10^6^	7.9 × 10^10^
Poisson’s ratio	0.3	0.3
Coefficient of restitution of soil—other	0.2	0.26
Coefficient of static friction of Soil—Other	0.54	0.5
Coefficient of rolling friction of Soil—Other	0.2	0.04

The 3D model of the slope was established by the SOLIDWORKS software and imported into the EDEM software as a pellet factory. The DEM particles were packed to a bulk voidage of 33.37*%* as measured for the soil in the field. Table 1 also lists input parameters used to define soil-soil and soil-tool interactions.

#### Contact model

The contact model is an important basis for analyzing the adhesion between mechanical parts and soil particles. During the digging operation, the soil particle is subjected to a variety of compound forces^24,25^. According to Newton’s second law, the linear motion and rotation equation of the soil particle p can be expressed as Eqs. (18)–(21). The Explanation of the symbols as show in “Table of Appendix”.

18 $${F}_{n,Pq}^{JKR}=-4\sqrt{\pi \gamma {E}^{*}{\xi }^\frac{3}{2}}+\frac{4{E}^{*}}{3{R}^{*}}{\xi }^{3}$$

19 $${F}_{coh,pq}={k}_{coh,pq}{A}_{coh,pq}$$

20 $${m}_{p}\frac{d{v}_{p}}{{d}_{t}}={m}_{p}g+{\sum }_{q=1}^{{n}_{p}}(-4\sqrt{\pi \gamma {E}^{*}{\alpha }^\frac{3}{2}}+\frac{4{E}^{*}}{3{R}^{*}}{\alpha }^{3}+{F}_{n,pq}^{d}+{F}_{\tau ,pq}+{F}_{\tau ,pq}^{d}+{k}_{coh,pq}{A}_{coh,pq})$$

21 $${I}_{p}\frac{d{\omega }_{p}}{{d}_{t}}=\sum_{q=1}^{{n}_{p}}\left({T}_{\tau ,pq}+{T}_{\gamma ,pq}\right)$$

The soil of afforestation land generally has a higher moisture content. Here there is cohesive and adhesive nature between the soil-soil and soil-tool. The cohesive force ${F}_{coh,pq}$ of soil particles is mainly set according to its internal cohesion characteristics. A Hertz–Mindlin with JKR and additional model-bounding contact model was adopted as the primary contact model for both particle–particle and particle–tool interactions. This model is suitable for simulating materials that have obvious adhesion and agglomeration between particles due to static electricity, moisture and other reasons. Table 2 lists the input parameters required for the contact models^26,27^.

**Table 2:** Parameters of contact model.

Parameter	Value
Normal stiffness per unit area	2.1 × 10^8^ N m^−3^
Shear stiffness per unit area	8 × 10^7^ N m^−3^
Critical normal stress	1.5 × 10^6^ Pa
Critical shear stress	8 × 10^5^ Pa
Bonded disk radius	2.5 mm
Surface energy of soil–soil	7.46 J m^−2^
Surface energy of soil–tool	5.5 J m^−2^

#### Scheme of simulation experiment

**Figure 6:**
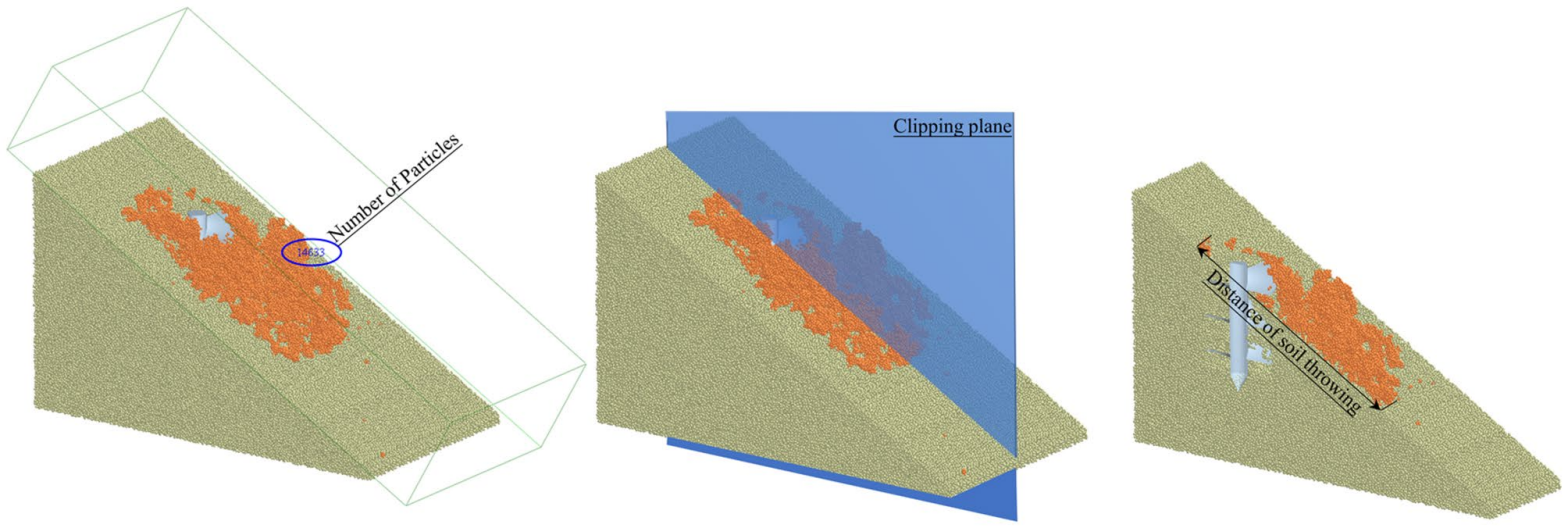
Acquisition of simulation indexes.

Virtual experiments on the quadratic rotating orthogonal center combination with four factors and five levels were carried out to evaluate the working performance of the auger. Based on previous experimental studies, practical experience and mechanism analysis, the appropriate levels of the experiment factors were established as indicated in Table 3. In actual production, some too steep and complex hillsides need to be prepared for land or soil. The slope angle was optimized to serve as a reference for the land preparation process. The slope angle *X*_1_, the helix angle of auger *X*_2_, the feeding speed *X*_3_ and the rotating speed of auger *X*_4_ were selected as experimental factors, while the efficiency of conveying-soil *Y*_1_ and the distance of throwing-soil *Y*_2_ set as experimental indicators. According to the accuracy in the practical application, the value in the virtual experiment retains two significant digits. As shown in Fig. 6, in the EDEM software analyst module, Grid Bin Group and Clipping Plane are added to measure the amount of soil outside the pit and the distance of throwing-soil.

**Table 3:** Factors and levels of virtual experiment.

Coded value	Experiment factors			
	X_1_/($^\circ$)	X_2_/($^\circ$)	X_3_/(m/s)	X_4_/(r/min)
2	45	22	0.1	120
1	40	19	0.085	97.5
0	35	16	0.07	75
− 1	30	13	0.055	52.5
− 2	25	10	0.04	30

## Results and discussion

### Experiment results and regression model

The simulation experiment results based on the design scheme are presented in Table 4, including 24 analysis factors and 7 zero-point experiments for estimating the errors. Quadratic multiple regression analysis of the results in Table 4 was performed using the Design-Expert software, and the regression models between the influencing factors and evaluation indices were established as follows:

$$ Y_{{1}} = {1767.57} - {64.29}X_{{1}} + {117.46}X_{{2}} + {324.46}X_{{3}} + {107.87}X_{{4}} - {21.81}X_{{1}} X_{{2}} + {17.94}X_{{1}} X_{{3}} - {41.44}X_{{1}} X_{{4}} + {16.69}X_{{2}} X_{{3}} - {41.19}X_{{2}} X_{{4}} + {73.56}X_{{3}} X_{{4}} + {23.2}{X_{{1}}^{{2}}} - {82.42}{{X_{{2}}}^{{2}}} - {13.17}{{X_{{3}}}^{{2}}} - {53.67}{{X_{{4}}}^{{2}}} $$

$$ Y_{{2}} = {1968.14} + {636.42}X_{1} + {34.42}X_{2} + {66}X_{3} + {115.17}X_{{4}} + {28.63}X_{{1}} X_{{2}} + {9.13}X_{{1}} X_{{3}} - { 45.87}X_{{1}} X_{{4}} + {1}0X_{{2}} X_{{3}} + {30.5}X_{{2}} X_{{4}} - {1.75}X_{{3}} X_{{4}} + {55.03}{X_{{1}}^{{2}}} - {8.1}{{X_{{2}}}^{{2}}} - {72.72}{{X_{{3}}}^{2}} + {61.03}{{X_{{4}}}^{{2}}} $$

**Table 4:** Experiment schemes and results.

No.	Factors				Evaluation indices	
	X_1_	X_2_	X_3_	X_4_	Y_1_	Y_2_
1	− 1	− 1	− 1	− 1	1243	1246
2	1	− 1	− 1	− 1	1143	2435
3	− 1	1	− 1	− 1	1572	1023
4	1	1	− 1	− 1	1330	2517
5	− 1	− 1	1	− 1	1502	1150
6	1	− 1	1	− 1	1672	2633
7	− 1	1	1	− 1	2039	1186
8	1	1	1	− 1	1973	2619
9	− 1	− 1	− 1	1	1376	1432
10	1	− 1	− 1	1	1096	2625
11	− 1	1	− 1	1	1469	1480
12	1	1	− 1	1	1378	2763
13	− 1	− 1	1	1	2111	1545
14	1	− 1	1	1	1926	2683
15	− 1	1	1	1	2351	1580
16	1	1	1	1	2006	2831
17	− 2	0	0	0	1971	1024
18	2	0	0	0	1769	3429
19	0	− 2	0	0	1255	1830
20	0	2	0	0	1640	2118
21	0	0	− 2	0	1021	1496
22	0	0	2	0	2428	1935
23	0	0	0	− 2	1225	2092
24	0	0	0	2	1900	2409
25	0	0	0	0	1780	1988
26	0	0	0	0	1800	1994
27	0	0	0	0	1822	2015
28	0	0	0	0	1870	2086
29	0	0	0	0	1676	1856
30	0	0	0	0	1689	1889
31	0	0	0	0	1736	1949

Refer to Table 3. *Y*_1_ for efficiency of conveying-soil. *Y*_2_ for distance of the throwing-soil. The number of factors in these experiments is m = 4. The asterisk arm is of length 2. ± 2 indicates the asterisk arm experiment code. ± 1 indicates the two-level experiment code. 0 indicates the zero-level experiment code. The number of asterisk arm experiments is 2 m = 8. The number of two-level experiments 2 m = 16. The number of experiments with zero-levels was at least one. And the number of experiments with zero-levels was empirically chosen to be seven.

**Figure 7:**
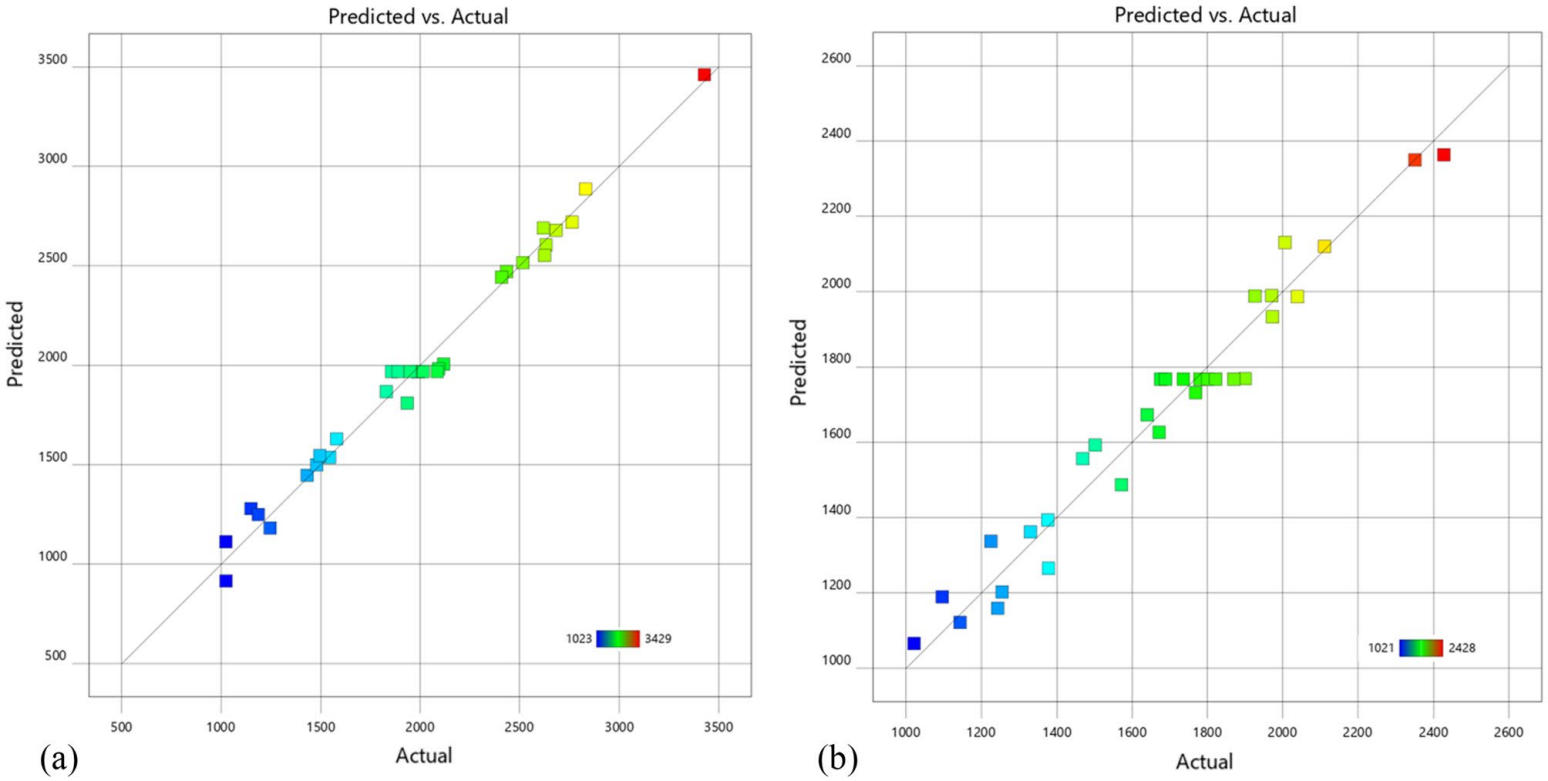
Scatter plot. (**a**) Scatter plot of actual and predicted distance of throwing-soil. (**b**) Scatter plot of actual and predicted efficiency of conveying-soil.

The relationship between the actual values of the efficiency of conveying-soil and the distance of throwing-soil and the predicted values of the regression model is shown in Fig. 7. It can be seen from Fig. 7 that the actual values are basically distributed on the predicted curve, consistent with the trend of the predicted values, and linearly distributed.

### Variance analysis and discussion

The F-test and analysis of variance (ANOVA) were performed on the regression coefficients in the regression models of the evaluation indices *Y*_1_ and *Y*_2_, and the results are shown in Table 5. According to the significance values *P* of the lack of fitting in the regression models of the objective functions *Y*_1_ and *Y*_2_ in Table 5, *PL*_1_ = 0.1485 > 0.05 and *PL*_2_ = 0.2337 > 0.05 (both were not significant), indicating that no loss factor existed in the regression analysis, and the regression model exhibited a high fitting degree.

**Table 5:** ANOVA results of regression model.

Indicator	Source of variance	Sum of squares	df	Mean square	F-value	p-value	Significant
*Y*_1_	Model	3.686E+06	14	2.633E+05	27.92	< 0.0001	***
	X_1_	99,202.04	1	99,202.04	10.52	0.0051	***
	X_2_	3.311E+05	1	3.311E+05	35.12	< 0.0001	***
	X_3_	2.527E+06	1	2.527E+06	267.97	< 0.0001	***
	X_4_	2.793E+05	1	2.793E+05	29.62	< 0.0001	***
	X_1_X_2_	7612.56	1	7612.56	0.8074	0.3822	Not significant
	X_1_X_3_	5148.06	1	5148.06	0.5460	0.4707	Not significant
	X_1_X_4_	27,473.06	1	27,473.06	2.91	0.1072	*
	X_2_X_3_	4455.56	1	4455.56	0.4726	0.5017	Not significant
	X_2_X_4_	27,142.56	1	27,142.56	2.88	0.1091	*
	X_3_X_4_	86,583.06	1	86,583.06	9.18	0.0080	***
	X_1_^2^	15,392.56	1	15,392.56	1.63	0.2196	Not significant
	X_2_^2^	1.943E+05	1	1.943E+05	20.60	0.0003	***
	X_3_^2^	4962.99	1	4962.99	0.5264	0.4786	Not significant
	X_4_^2^	82,381.76	1	82,381.76	8.74	0.0093	***
	Residual	1.509E+05	16	9428.56			
	Lack of Fit	1.206E+05	10	12,064.13	2.40	0.1485	Not significant
	Pure Error	30,215.71	6	5035.95			
	Cor Total	3.837E+06	30				
Y_2_	Model	1.062E+07	14	7.586E+05	81.87	< 0.0001	***
	X_1_	9.721E+06	1	9.721E+06	1049.03	< 0.0001	***
	X_2_	28,428.17	1	28,428.17	3.07	0.0990	*
	X_3_	1.045E+05	1	1.045E+05	11.28	0.0040	***
	X_4_	3.183E+05	1	3.183E+05	34.35	< 0.0001	***
	X_1_X_2_	13,110.25	1	13,110.25	1.41	0.2516	Not significant
	X_1_X_3_	1332.25	1	1332.25	0.1438	0.7095	Not significant
	X_1_X_4_	33,672.25	1	33,672.25	3.63	0.0747	*
	X_2_X_3_	1600.00	1	1600.00	0.1727	0.6833	Not significant
	X_2_X_4_	14,884.00	1	14,884.00	1.61	0.2232	Not significant
	X_3_X_4_	49.00	1	49.00	0.0053	0.9429	Not significant
	X_1_^2^	86,586.40	1	86,586.40	9.34	0.0075	***
	X_2_^2^	1875.34	1	1875.34	0.2024	0.6588	Not significant
	X_3_^2^	1.512E+05	1	1.512E+05	16.32	0.0009	***
	X_4_^2^	1.065E+05	1	1.065E+05	11.49	0.0037	***
	Residual	1.483E+05	16	9266.33			
	Lack of fit	1.119E+05	10	11,190.65	1.85	0.2337	Not significant
	Pure error	36,354.86	6	6059.14			
	Cor total	1.077E+07	30				

***Means extremely significant (P < 0.01); **Means very significant(0.01 ≤ P < 0.05); *Means significant(0.05 ≤ P < 0.1). “df” means degree of freedom.

According to the ANOVA, the significance values *P* of each influencing factor in the test could be determined^28^. For the evaluation index Y_1_, the factors *X*_1_, *X*_2_, *X*_3_, *X*_4_, *X*_3_*X*_4_, *X*_2_^2^, *X*_4_^2^ had extremely significant influences, while the factors X_*1*_X_*4*_, X_*2*_X_*4*_ had a significant influence. For the evaluation index *Y*_2_, the factors *X*_1_, *X*_3_, *X*_4_, *X*_1_*X*_4_, *X*_1_^2^, *X*_3_^2^, *X*_4_^2^ had extremely significant influences, and the factors *X*_2_, *X*_1_*X*_4_ had a significant influence. Within the level range of the selected factors, according to the *F* value of each factor as shown in Table 5, the weight of the factors affecting the efficiency of conveying-soil is feeding speed > helix angle of auger > rotating speed of auger > slope angle. And the weight of the factors affecting the distance of throwing-soil is slope auger > rotating speed of auger > feeding speed > helix angle of auger.

In addition, it is obvious that there are interactions between the feeding speed and rotating speed of the auger, slope auger and rotating speed of auger, helix angle of the auger and rotating speed of the auger on the efficiency of conveying-soil *Y*_1_. For the distance of throwing-soil *Y*_2_, there is an interaction between the slope angle and the rotating speed of the auger.

### Analysis of response surface

The fitting coefficient of the efficiency of conveying-soil is R^2^ = 0.9714, R^2^_adjust_ = 0.9263, R^2^_pred_ = 0.8082, the difference between R^2^_adjust_ and R^2^_pred_ is less than 0.2. The fitting coefficient of the distance of throwing-soil is R^2^ = 0.9873, R^2^_adjust_ = 0.9742, R^2^_pred_ = 0.9355, the difference between R^2^_adjust_ and R^2^_pred_ is smaller than 0.2. It is indicated that the response surfaces of the two models established have good consistency and predictability for the experimental results^29^.

**Figure 8:**
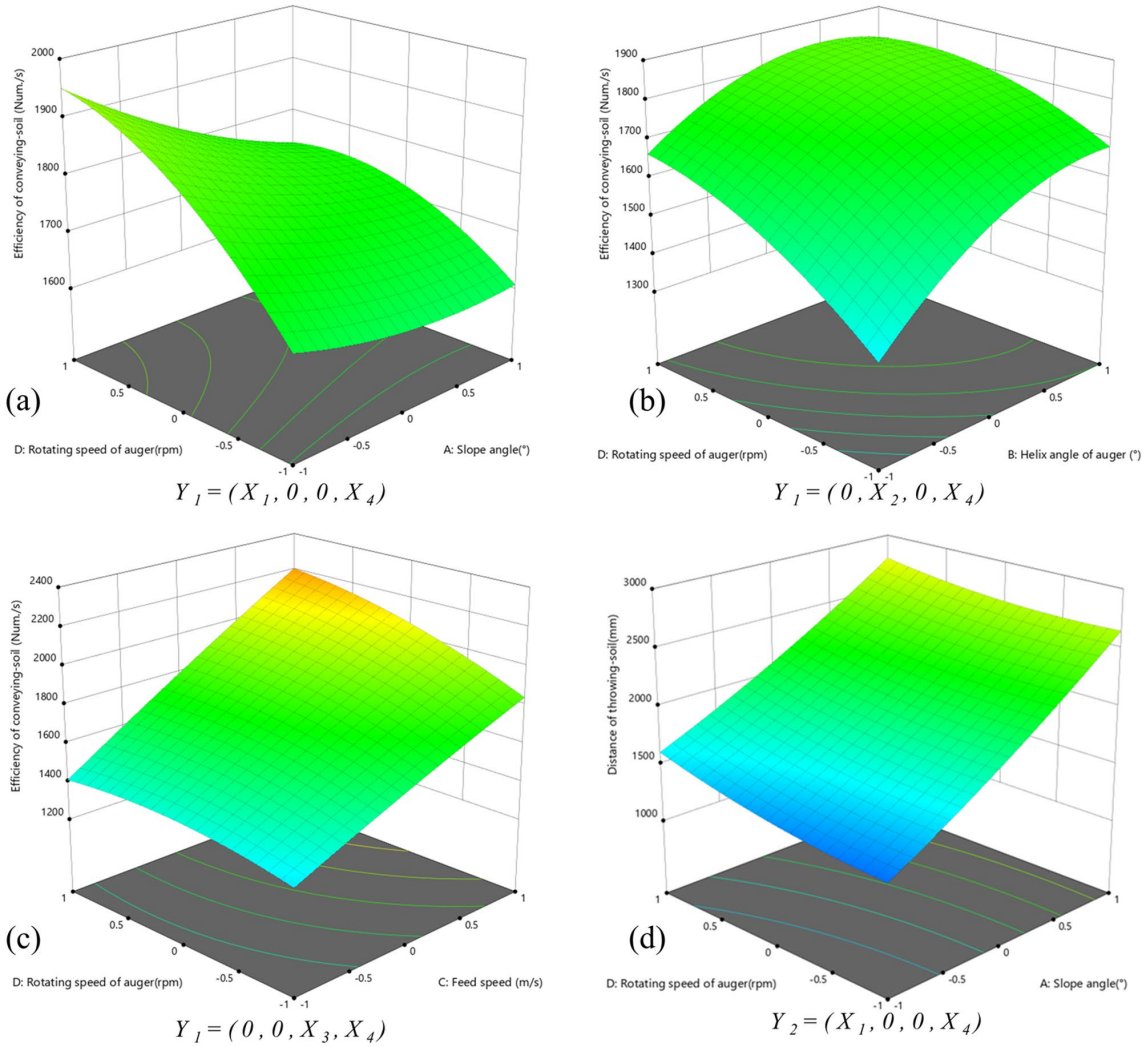
3D response diagram effect of evaluation indices. (**a**) Effect of interaction between *X*_*1*_ and *X*_*2*_ on efficiency of conveying-soil. (**b**) Effect of interaction between *X*_*2*_ and *X*_*4*_ on efficiency of conveying-soil. (**c**) Effect of interaction between *X*_*3*_ and *X*_*4*_ on efficiency of conveying-soil. (**d**) Effect of interaction between *X*_*3*_ and *X*_*4*_ on distance of throwing-soil.

The response surface is created directly using the Design-Expert software. After entering the data, select “Analysis” module. In the “Model-Graph” menu bar, select “3D-surface” to switch to the 3D view. To express the interactive influence of each factor on the efficiency of conveying-soil *Y*_1_ and distance of the throwing-soil *Y*_2_, the above two quadratic regression equations of the evaluation indices were subjected to the dimensionality reduction treatment. Two of the factors was set to level 0, while the other two underwent interaction effect analysis to study the influence law on the evaluation indices *Y*_1_ and *Y*_*2*_, and the corresponding response surfaces were generated, as illustrated in Fig. 8.

It can be seen in Fig. 8a, when the slope angle was constant, the efficiency of conveying-soil increased with the rotating speed of the auger to a certain value, then the efficiency increase changed more gently. The reasons for this phenomenon are described as follows. On the one hand, the greater the kinetic energy of the soil when leaving the original position, and the thinner the soil was cut, resulting in the smaller the probability of blockage in the spiral blade space. On the other hand, the centrifugal force of soil arriving at the pit mouth is greater, so it does not obstruct in the pit mouth. However, if the rotation speed of the auger was too high and the soil layer cut was too thin, the subsequent soil's driving effect to the front would be weakened, or even the flow would be interrupted, so the vertical rising speed of the soil would be reduced. When the rotational speed of the auger was constant, the efficiency of conveying-soil decreased with the increase of slope and then slightly increased. With the increase of slope, the time of slope cutting process increased, and there was more soil backfilling on the side of high altitude, which leaded to the reduction of soil discharge efficiency. However, with the increase of slope, the amount of soil slide at the pit mouth was increased, improving the efficiency of soil discharge. Further analysis demonstrated that the response surface for *Y*_1_ changed more rapidly in the direction of the rotating speed than in that of the slope angle, indicating that the rotating speed of auger *X*_4_ had a more significant influence than the slope angle *X*_1_.

As can be seen in Fig. 8b, when the helix angle of the auger was fixed, the efficiency of conveying-soil continued to increase with the increase of the rotation speed. When the rotating speed of auger was fixed, the efficiency of conveying-soil increased with the increase of the helix angle and tends to decrease when it reached a certain value. The spiral blades space was the channel of soil movement. This phenomenon was caused by the increase of the gap between the two spiral blades with the increase of the helix angle of the auger, the soil was not easy to produce blockage. Meanwhile, the movement distance of soil was shorter, and the soil with higher kinetic energy was discharged more quickly from the pit. When reaching the pit mouth, the angle of soil throwing was larger and the soil backfilling rate was reduced. However, if the helix angle of auger was too large, the upward support ability and friction of the spiral blade surface to the soil would be reduced. Further analysis demonstrated that the response surface for *Y*_1_ changed more rapidly in the direction of the helix angle than the rotating speed of the auger, indicating that the helix angle of the auger *X*_*2*_ had a more significant influence than the rotating speed of the auger *X*_4_.

When the feeding speed was fixed, the efficiency of throwing-soil continued to increase with the increase of the rotating speed. When the rotating speed of auger was fixed, the efficiency of the throwing-soil with the increase of the feeding speed (see in Fig. 8c). The phenomenon was caused by the faster the feeding speed of the auger, the thickness of soil cut per unit time increased. Furthermore, the subsequent driving force of soil increased, and the soil kinetic energy increased. However, in the actual production, excessive feeding speed would cause soil blockage on the surface of spiral blades. The reason is due to in the simulation process, the soil would not stop moving because of blockage. Further analysis demonstrated that the response surface for *Y*_1_ changed more rapidly in the direction of the rotating speed than in that of the feeding speed, indicating that the rotating speed of auger *X*_4_ had a more significant influence than the feeding speed *X*_3_.

When the slope was fixed, the distance of the throwing-soil increased with the increase of rotation speed of the auger, and the increase amplitude increased gradually, as shown in Fig. 8d. The reason for this phenomenon was that the soil had more kinetic energy when it left its original position and the centrifugal force it received when it reaching the pit mouth is greater. When the rotation speed was too low, the soil layer was thin and the subsequent soil driving force was insufficient, resulting in the soil mass per unit area at the pit mouth was light and then the kinetic energy was small. When the rotating speed of auger was fixed, the distance of the throwing-soil increased continuously with the increase of the slope. As the slope increased, the time of soil swipe down process increased and then the rolling distance on the slope increased. Further analysis demonstrated that the response surface for *Y*_2_ changed more rapidly in the direction of the slope angle than in that of the rotating speed of auger, indicating that the slope angle *X*_1_ had a more significant influence than the rotating speed *X*_3_.

### Comprehensive optimal design

As relative importance and influencing rules of various experimental factors on evaluation indexes were different from each other, evaluation indexes should be taken into comprehensive consideration^30^. The optimization equation is obtained by the Design-Expert software multi-objective optimization method with *Y*_1_ and *Y*_2_ as the optimization objective function.

$$25\le {X}_{1}\le 45$$

$$10\le {X}_{2}\le 22$$

$$0.04\le {X}_{3}\le 0.1$$

$$30\le {X}_{4}\le 120$$

$${{Y}_{1}}_{\mathrm{max}}({X}_{1},{X}_{2},{X}_{3},{X}_{4})$$

$${{Y}_{2}}_{min}({X}_{1},{X}_{2},{X}_{3},{X}_{4})$$

In practice, the best combination of parameters needs to be selected according to the terrain slope. When the slope was fixed, the Design-Expert software was applied to optimize and solve the above mathematical model. The optimal combination of working parameters affecting the efficiency of conveying-soil *Y*_1_ and distance of throwing-soil *Y*_2_ for the auger were obtained and are shown in Table 6. If the ground preparation was required before the digging operation, the digging parameters can be designed according to values of Group 6 in Table 6.

**Table 6:** Optimal parameter combinations of several terrain slopes.

No.	Slope (°)	Helix angle (°)	Feeding speed (m/s)	Rotating speed (r/min)	Efficiency of conveying-soil (Num/s)	Distance of throwing-soil (mm)
1	25	21.371	0.094	91.639	2622.162	997.673
2	30	20.863	0.1	58.091	2274.818	1108.703
3	35	15.563	0.1	69.303	2270.547	1777.915
4	40	10	0.1	85.486	1897.109	2335.958
5	45	10	0.1	85.379	1961.625	3076.999
6	26.467	21.567	0.1	67.408	2450.607	762.168

### Disturbance of soil

**Figure 9:**
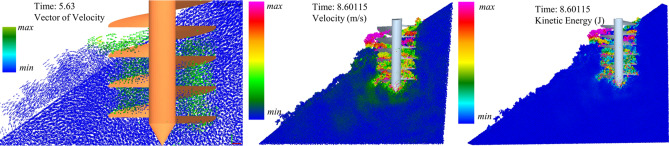
The disturbance of the soil effect by spiral blade.

A soil disturbance is defined as the loosening, movement and mixing of soil caused by an auger passing through the soil^16^. In the interface of the EDEM Analyst, add a “Clipping plane” to show the movement of the auger inside the pit. The kinetic energy, soil particle velocity vector, and velocity value of soil particles is observed when the auger in the middle of the soil bin^31,32^, as shown in Fig. 9.

The soil was lifted to the surface and then dropped to the lower side. In addition to the volume occupied by the spiral blades, the disturbed area also included the out-of-pit disturbed area caused by the compression of the cutting end of the spiral blade, as shown in the lower left corner of the auger.

The kinetic energy and velocity of soil decreased firstly and then increased along the opposite direction of the auger feeding. The cutting end of the auger and the soil-throwing section occurred in the region with high kinetic energy and velocity. This was because the maximum kinetic energy was obtained at the cutting end of the auger, which was gradually consumed in the process of rising. After reaching the dumping end, the soil lost the restraint of the pit wall. When the centrifugal force of soil lost the reaction force, the kinetic energy of soil increased. Too much kinetic energy, however, can cause the soil to spread too far, causing subsequent trouble. The kinetic energy of the soil at the cutting end was related to the rotational speed of the auger. The spiral angle affected the angle between the force and gravity, and then the kinetic energy consumption in the process of soil increased.

### Verification experiments

**Figure 10:**
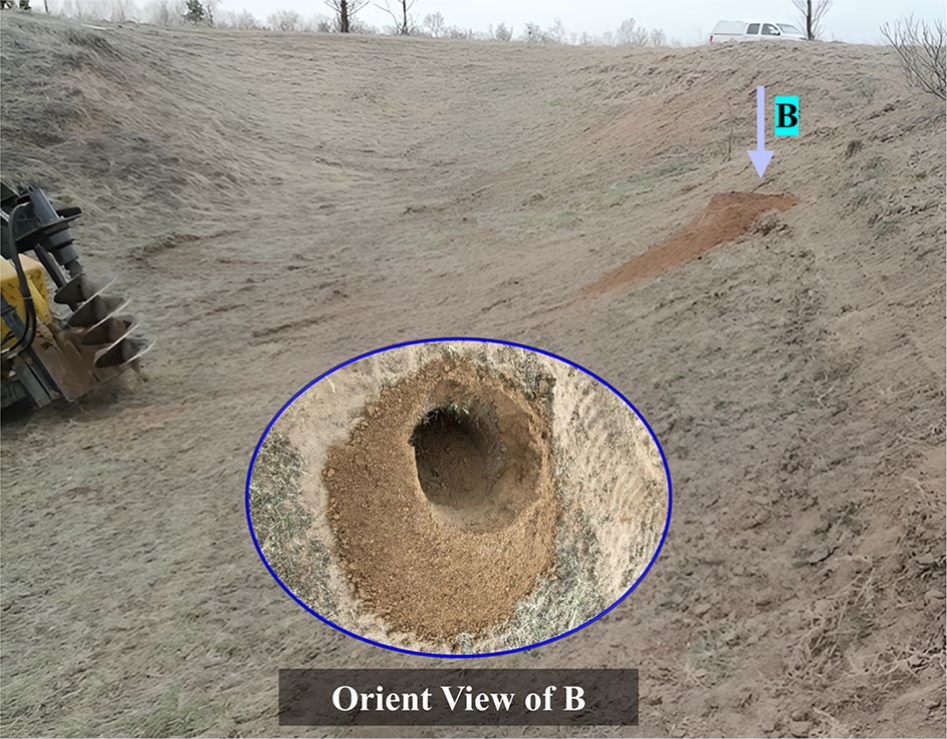
Operation diagram at the experiment site.

To verify the accuracy of the optimization model for auger working, as well as to evaluate the rationality of the working parameter combination optimized by the virtual experiment, performance verification tests were carried out on the EDEM software. According to the optimized process parameter setting test (as shown in Table 6), the relative error between the theoretical value and the experimental value was obtained. The verification test results are summarized in Table 7. The average relative errors of the efficiency of conveying-soil and the distance of throwing-soil between the Theoretical value and text value were only 4.4%, 9.1%. The simulation model is fairly accurate. The field performance verification experiments were carried out in slope. Figure 10 illustrates the field test and working conditions.

**Table 7:** Results and comparison of validation test.

Text	Efficiency of conveying-soil (Num/s)		Relative error (%)	Distance of throwing-soil (mm)		Relative error (%)
	Theoretical value	Text value		Theoretical value	Text value	
1	2622	2739	4.4	998	1025	2.8
2	2275	2327	2.3	1109	1187	7.0
3	2271	2221	2.2	1778	1689	5.0
4	1897	1945	2.5	2336	2769	18.5
5	1962	1763	10.1	3077	3166	2.9
6	2451	2576	5.1	762	900	18.1
Average	/	/	4.4	/	/	9.1

## Conclusions

This paper aims at the mechanism and method of constructing fish-scale pit in hilly regions. Improve work efficiency and performance. The soil and slope modeling are developed using EDEM, and the process of auger cutting and transporting soil on slope is simulated. Through the simulation results, the dynamic characteristics of soil are analyzed, and the structural parameters and operation parameters of auger are optimized.

In the process of digging pits in hilly regions to assist in the construction of fish-scale pits:

1. The performance of auger working on slope can be evaluated by monitoring the efficiency of conveying-soil and the distance of the throwing-soil.
2. The weight of the factors affecting the efficiency of conveying-soil is feeding speed > helix angle of auger > rotating speed of auger > slope angle. The weight of the factors affecting the distance of throwing-soil is slope auger > rotating speed of auger > feeding speed > helix angle of auger.
3. According to the optimization results, the optimal parameter combination can be obtained in different slope operations. If the land preparation is required before the digging operation, the optimal slope angle is about 26°.
4. Compared with the plain area, the variation law of soil displacement and velocity is different in hilly regions. The errors between the results from the developed DEM simulation modeling and virtual experiments results are in the acceptable accuracy, confirming the effectiveness of the DEM model for estimating the working efficiency of the earth auger in hilly area.

## Supplementary Information

Supplementary Table 1.

## Data Availability

The data that support the findings of this study are available from the corresponding author.

## References

[1] Yu JG, Qu JW (2006). Current research situation and development trend of earth auger in home and abroad. J. Agric. Mech. Res..

[2] Fang Q (2020). Application of excavator in forestry development. Agric. Sci. Technol. Equip..

[3] Jin C (2021). Development of earth augers and application in stony mountain regions. For. Mach. Wood Work. Equip..

[4] Yang Z (2013). Performance test of hand-held electric hole-digger for fertilization in orchard. Trans. CSAE.

[5] Wang K (2020). Combining infiltration holes and level ditches to enhance the soil water and nutrient pools for semi-arid slope shrubland revegetation. Sci. Total Environ..

[6] Feng TJ, Wang W (2019). Combining land preparation and vegetation restoration for optimal soil eco-hydrological services in the Loess Plateau, China. Sci. Environ..

[7] Feng TJ (2016). Effects of land preparations and vegetation types on soil chemical features in a loess hilly region. Acta Ecol. Sin..

[8] Zheng JY (2019). Fish-scale pits with infiltration holes enhance water conservation in semi-arid loess soil: Experiments with soil columns, mulching, and simulated rainfall. J. Soil Sci. Plant Nutr.

[9] Zhuo FY (1989). Digging Machine.

[10] Lian SH, Li ZM (1975). Theoretical analysis of soil-raising on the drill bit of a digger. Grain Oil Process. Food Mach..

[11] Macpherson JD, Jogi PN, Kingman JEE (2001). Application and analysis of simultaneous near bit and surface dynamics measurements. SPE Drill. Complet..

[12] Purtskhvanidze M, Keller N (1990). Hole digger for slopes. Sel'skiĭ Mekhanizator.

[13] Yang Y, Liang SM, He YB (2021). Research and analysis of a portable digging machine. Mach. Des. Manuf..

[14] Lou YY, Liu GH (2017). The analysis of the structure and properties of new banana digging robot. J. Agric. Mech. Res..

[15] Yang WZ, Huang YG, Wang CP (2012). Dynamics analysis and simulation of the earth auger actuator. J. Northw. For. Univ..

[16] James Barr, J. F. Discrete element modelling of narrow point openers to improve soil disturbance characteristics of no-till seeding systems. in *2016 ASABE Annual International Meeting Sponsored by ASABE* (2016).

[17] Aikins KA (2021). Analysis of effect of bentleg opener geometry on performance in cohesive soil using the discrete element method. Biosyst. Eng..

[18] Wang JW (2019). Optimization design and experiment of the rotary tillage directional soil-collecting device of unilateral ridger for paddy field. Int. Agric. Eng. J..

[19] Viktor M, Lars JM, Ying C, Nyord T (2018). Modelling approach for soil displacement in tillage using discrete element method. Soil Tillage Res..

[20] Jin Y (2021). Design and experiment of in-situ fertilizer mixing integrated digging and backfilling planter for fruit tree. Trans. Chin. Soc. Agric. Mach..

[21] Ma L (2017). Design and experiment of automatic feed mechanism of the portable digging machine. Trans. CSAE.

[22] Alessandro M, Stefano M (2021). A flexible multi-body model of a surface miner for analyzing the interaction between rock-cutting forces and chassis vibrations. Int. J. Min. Sci. Technol..

[23] Sun JY (2018). DEM simulation of bionic subsoilers (tillage depth > 40 cm) with drag reduction and lower soil disturbance characteristics. Adv. Eng. Softw..

[24] Wang JW (2017). Numerical analysis and performance optimization experiment on hanging unilateral ridger for paddy field. Trans. CSAM.

[25] Wang YC (2021). Study on Mining Mechanism of Panax Noto Ginseng Based on Discrete Element Method.

[26] Tong ZW (2020). Design and Experiment of Compound Cutting Parts of Tobacco Hilling Machine.

[27] Li JW (2019). Calibration of parameters of interaction between clayey black soil with different moisture content and soil-engaging component in northeast China. Trans. CSAE.

[28] Shi YY (2019). Numerical simulation and field tests of minimum-tillage planter with straw smashing and strip laying based on EDEM software. Comput. Electron. Agric..

[29] Sun JF (2020). Study on plowing performance of EDEM low-resistance animal bionic device based on red soil. Soil Tillage Res..

[30] Li LQ, Wang DF, Xing Y (2018). Study on round rice straw bale wrapping silage technology and facilities. Int. J. Agric. Biol. Eng..

[31] Yong-Jae S (2020). Interacting analysis between wheel and sand particles based on DEM and its validation with experiments. J. Mech. Sci. Technol..

[32] Il-Kab J (2019). Effect of crushing conditions of crushing rate in process analysis of sewage-sludge organic solid-fuel crusher using the discrete element method. J. Mech. Sci. Technol..

