# Supplementary material for: Numerical simulation and parameter optimization of earth auger in hilly area using EDEM software
Source: Sci Rep. 2022 Nov 14;12:19526. doi: 10.1038/s41598-022-23833-2 (PMC9663533; doi:10.1038/s41598-022-23833-2)
Supplement: Supplementary file 1 — Supplementary Table 1. [file 41598_2022_23833_MOESM1_ESM.docx]

| Symbol | Explanation of the symbols |
| --- | --- |
| *v_0_* | The absolute velocity of the soil as it leaves the spiral blade |
| *t* | Time |
| *m* | The mass of soil |
| *r* | The radius of auger |
| *ω* | The angular velocity of auger |
| *v_r_* | The vertical velocity of soil |
| *ꞵ* | The helix angle of auger |
| *N_i_* | The force of friction on soil particles |
| *μ* | The coefficient of friction. |
| *r_0_* | The radius of rod |
| *φ* | The Angle of rotation of soil motion |
| *f_1_* | The driving force of subsequent soil |
| *f_2_* | The resistance from the soil ahead |
| *v_a_* | The vertical velocity of soil movement |
| *A, B, C, E, P* | The constant coefficients |
| $v_{1}^{x}$, $v_{1}^{y}$ | The horizontal and vertical velocity of soil during the up-projectile motion. |
| $v_{2}^{x}$, $v_{2}^{y}$ | The horizontal and vertical velocity of soil during the downward-projectile motion |
| *k* | The coefficient of air resistance |
| *g* | The acceleration of gravity |
| *α* | angle of slope |
| ${pos}_{1}^{x}$, ${pos}_{1}^{y}$ | The soil displacements in the horizontal and vertical directions during the up-projectile motion |
| ${pos}_{2}^{x}$, ${pos}_{2}^{y}$ | The soil displacements in the horizontal and vertical directions during the downward projectile motion |
| *Δx* | The soil displacements during the soil slides down on the slope |
| *v*_r_ | The velocity of the soil relative to the surface of the blade |
| *Q*_0_ | The volume of the original soil in pit |
| *Q*_1_ | The volume of soil cut by auger after turning the angle *φ* |
| *Q*_2_ | The volume of soil discharged at the discharge end of the auger *φ* |
| *Q*_3_ | The space where soil can be stored on the auger |
| $K_{1},K_{2},K_{3}$ | The bulk coefficients of soil at this position |
| *h* | The thickness of cut down the soil after turning the angle *φ* |
| *H* | The total depth of pit |
| *d* | The thickness of the spiral blade |
| $F_{n,Pq}^{JKR}$ | The normal contact force between particles or between particles and equipment parts |
| $F_{n,pq}^{d}$ | The normal damping |
| $F_{\tau,pq}$ | The tangential contact force |
| $F_{\tau,pq}^{d}$ | The tangential damping |
| $F_{coh,p q}$ | The internal particle adhesion |
| $I_{p}$ | The moment of inertia of the soil particle $p$ |
| $n_{p}$ | The oil particles$p$ total number of particles in contact |
| $\omega_{p}$ | The rotational angular velocity of the soil particle $p$ |
| $T_{\tau,pq}$ | The tangential moment of the soil particle *p* |
| $T_{\gamma,pq}$ | The rolling moment of the soil particle $p$; |
| $k_{coh,pq}$ | The soil particle adhesion energy density; |
| $A_{coh,pq}$ | The soil particle contact area; |
| $\gamma$ | The surface energy; |
| $E^{*}$ | The equivalent Young's modulus |
| $R^{*}$ | The equivalent radius |
| $\xi$ | The interaction parameter |

**Table of Appendix.** Nomenclature
